# Neurobiological basis of autism spectrum disorder: mini review

**DOI:** 10.3389/fpsyg.2025.1558081

**Published:** 2025-05-30

**Authors:** Maria Vanessa Freitas Holanda, Eva da Silva Paiva, Larissa Nayara de Souza, Karina Maia Paiva, Rodrigo Freire Oliveira, Élyssa Adriolly Freitas Tavares, Paulo Leonardo Araújo de Góis Morais, Ariel Moraes de Andrade, Maria Irany Knackfuss, Ellany Gurgel Cosme do Nascimento, José Rodolfo Lopes de Paiva Cavalcanti

**Affiliations:** ^1^Graduate Program in Health and Society (PPGSS) at the State University of Rio Grande do Norte, Mossoró, Brazil; ^2^Multicenter Graduate Program in Biochemistry and Molecular Biology (PMBqBM) at the State University of Rio Grande do Norte, Mossoró, Brazil; ^3^Multicenter Graduate Program in Physiological Sciences (PPGMCF) at the State University of Rio Grande do Norte, Mossoró, Brazil

**Keywords:** autism spectrum disorder, neurobiological bases, morphological changes, genetic aspects, neurodevelopmental disorder

## Abstract

Autism Spectrum Disorder is a neuropsychiatric condition characterized by deficits in communication, social interaction, and repetitive behaviors, with significant symptom variability. This multifaceted profile reflects a complex genetic architecture as well as diversity in morphological characteristics. Therefore, the objective of this review is to discuss the genetic and morphological aspects that may contribute to understanding autism. No temporal restrictions were applied for study inclusion, nor were there limitations regarding language. Scientific articles available in full text and directly related to the topic were included, while editorials, letters, conference abstracts, theses, dissertations, and books were excluded. The results of this review converge on two main aspects: (1) genetic and morphological findings are essential for a more comprehensive understanding of the disorder, providing an important basis for investigating its underlying mechanisms; and (2) despite their relevance, the results are still incipient and insufficient to explain the full clinical and behavioral heterogeneity associated with autism, highlighting the need for further studies.

## 1 Introduction

Autism Spectrum Disorder (ASD) is classified by the *Diagnostic and Statistical Manual of Mental Disorders (DSM-5)* as a neurodevelopmental disorder, characterized by significant difficulties in communication and social interaction, along with restrictive and repetitive patterns of behavior (American Psychiatric Association, 2021).

Autism was first described in 1943 by child psychiatrist Leo Kanner, who studied the disorder while following 11 children. Since then, experts have debated whether ASD is an innate or acquired condition. For years, the prevailing view was that environmental factors were the primary determinants (Hyman et al., 2020). Later, research shifted to genetics, indicating a strong genetic contribution, with higher concordance rates among monozygotic twins (Hallmayer et al., 2011).

Changes in ASD diagnostic criteria unified autism-related diagnoses and now require a specific combination of difficulties in socialization, communication, and repetitive behaviors (Matson and Kozlowski, 2011). This growth is attributed mainly to changes in diagnostic criteria and greater awareness of the disorder (Thapar and Rutter, 2021). Moreover, the search for objective measures, such as biomarkers, aims to support early ASD identification, considering the increasing need for earlier diagnoses and interventions (Levin and Nelson, 2015).

Brain morphology in children with autism may include characteristics such as increased brain volume, especially in the left hemisphere, as well as variations in gray and white matter, reflecting the complexity of neural development associated with the disorder. These aspects are essential for understanding the neurobiological basis of autism and its implications for behavior and cognitive functioning (Lamanna and Meldolesi, 2024).

Within this context, the present study will discuss some genetic and morphological alterations associated with ASD, exploring how these changes may contribute to the brain alterations observed in the disorder. The relationship between these alterations will also be addressed, with a focus on how genetic modifications may influence brain changes.

## 2 Morphological characteristics found in the brain of individuals with ASD

Brain development is a continuous process involving significant changes in structure and function. Understanding these changes is essential for the early identification of disorders such as ASD (Xie et al., 2023). Although the heterogeneity of autism poses a clinical challenge, brain alterations can serve as biomarkers for diagnosing and understanding this complex disorder (Varcin and Nelson, 2016).

Individuals with autism often exhibit excessive brain volume growth in the first years of life, followed by a slowdown in childhood and, in some cases, a decline during adolescence and adulthood (Courchesne et al., 2011). In a longitudinal MRI study, children diagnosed with ASD showed a generalized increase in cortical volume at 2 years of age. Additionally, autistic children exhibited significantly larger volumes of gray and white matter. The study’s authors indicated that excessive brain growth predominantly occurs in the first months after birth (Hazlett et al., 2011).

Previous studies have supported the hypothesis of increased cortical volume in children with autism; however, no corresponding increase was observed in cerebellar volume. Furthermore, the head circumference of these children appears normal at birth but begins to grow at an accelerated rate around 12 months of age (Hazlett et al., 2005).

MRI studies demonstrate that, despite initial brain volume growth, a decline occurs during adolescence and early adulthood (Roy and Uddin, 2021). One hypothesis to explain this excessive brain volume growth suggests it may result from a greater number of neurons and synaptic connections formed during the prenatal period. These atypical brain development alterations are considered linked to the clinical characteristics of ASD (Courchesne et al., 2011).

Peterson et al. (2021) suggest that although there is an initial increase in extra-axial cerebrospinal fluid (CSF) volume in autistic children, this volume tends to normalize over time, especially after 4 years of age. The authors propose that this normalization may be related to ongoing brain maturation during adolescence and adulthood.

**Figure 1:**
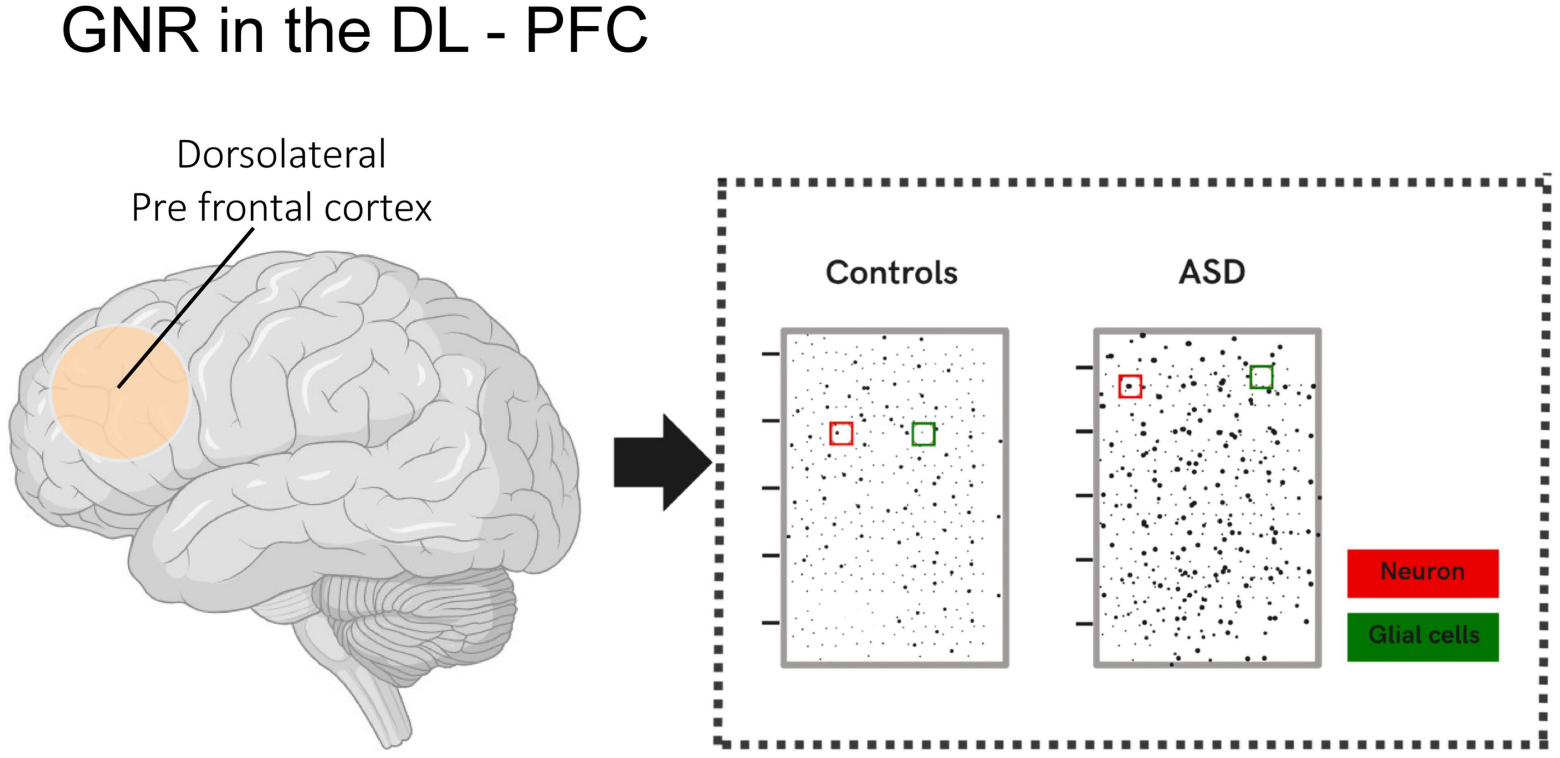
Illustration showing the glia-to-neuron ratio (GNR) in the DL-PFC of children with ASD and the control group. Changes in the glia-to-neuron ratio (GNR) in the DL-PFC were analyzed in children with ASD and compared to neurotypical children. The analysis revealed that the GNR was, on average, 20% lower in the DL-PFC of children with ASD. This result indicates a relative reduction in the number of glial cells, a proportional increase in the number of neurons per unit area, or both.

In a study conducted by Rabelo et al. (2023), cortical disorganization patches were identified in the dorsolateral prefrontal cortex (DL-PFC) of children with ASD and control groups. These patches showed disrupted gene expression, such as CALB1, RORB, and PCP4, in specific layers, particularly layers II-III. These patches exhibited a significantly reduced glia-to-neuron ratio (GNR) compared to unaffected regions and neurotypical brains. Additionally, the study revealed that the GNR in the DL-PFC of children with ASD was, on average, 20% lower, suggesting either a relative reduction in glial cells or an increased number of neurons. An increased proportion of neurons in the prefrontal cortex of individuals with ASD has also been reported in other studies, such as that of Falcone et al. (2021) (Figure 1).

Stoner et al. (2014) conducted an analysis of post-mortem samples from 22 brains, 11 of which belonged to children with ASD and 11 to controls. The study compared the organization of cortical layers between groups. The results revealed that 10 out of 11 cases showed areas of disorganization in the neocortex, indicating failures in neuronal migration during fetal development. These anomalies suggest that autism-associated alterations may originate in utero, impacting brain connectivity and cognitive, social, and linguistic functions.

In a parallel study, the impact of autism-associated genes on the connectivity between different cortical regions was analyzed. It was found that genetic alterations in ASD are more pronounced in the superficial layers of the cortex. Moreover, mutations in genes related to ASD are associated with interhemispheric communication, which may impair the integration of information from different areas of the brain (Parikshak et al., 2013).

Additionally, a study by Mei et al. (2023), which used a multimodal analysis of autistic subjects and controls, identified alterations in gray matter (GM) volume in various regions. A reduced GM volume was observed in areas such as the insula, inferior frontal gyrus, and orbitofrontal cortex, while an increased volume was noted in areas such as the calcarine cortex and middle frontal gyrus. The authors correlated these alterations with the behavioral profile of ASD.

These morphological and functional findings are increasingly understood through advances in genetics. Indeed, genetic research has revealed how certain mutations and genetic variants influence brain development and organization, contributing to the anomalies observed in brain structure and connectivity (Nalin et al., 2022).

## 3 Genetic bases of ASD

The relationship between genetics and psychiatric disorders is complex, and the identification of individual genes and the definitive evaluation of their effects remain persistent challenges. Twin studies have contributed to clarifying genetic influence, showing that identical twins are more likely to be diagnosed with ASD than fraternal twins (Colvert et al., 2015; Tick et al., 2016; Lichtenstein et al., 2010).

From this perspective, the etiology of ASD involves a complex interaction between genetic and environmental factors, modulated by epigenetic processes (Genovese and Butler, 2023). A study conducted by Bai et al. (2019) with 22,156 children in five countries identified that genetics accounts for approximately 80% of cases. Moreover, more than 800 genes and genetic syndromes associated with ASD have been identified, highlighting pathways such as chromatin remodeling and Wnt and Notch signaling, which affect brain development and neuroplasticity. Chromatin remodeling involves changes in the DNA and histone structure without altering the sequence itself, regulating gene expression and impacting neuronal differentiation and synapse formation (Genovese and Butler, 2023).

In this context, ASD can be classified into two forms: syndromic and non-syndromic. Syndromic ASD refers to the presence of specific genetic mutations that manifest as recognizable neurological syndromes, such as fragile X syndrome, Rett syndrome, and tuberous sclerosis (Nóbrega et al., 2024). On the other hand, non-syndromic ASD, also referred to as idiopathic, accounts for most cases and is not associated with other known neurological syndromes, although it involves alterations in genes related to autism (da Cruz Fontes and de Souza, 2022).

Inherited and *de novo* genetic mutations help provide important explanations for the disorder. Inherited mutations are passed from parents to offspring, while de novo mutations occur in the germline of a new generation. It is important to note that mutations are not necessarily harmful there are neutral or beneficial mutations, in addition to those that may lead to biological dysfunctions, often associated with neurodevelopmental disorders (Goldmann et al., 2019).

Rare and high-impact mutations, especially those affecting genes involved in chromatin remodeling and cell signaling, have been recognized as critical factors in the predisposition to ASD. These genetic changes cause significant morphological changes in the brain, including increased brain volume particularly in the prefrontal córtex abnormalities in dendritic spine density and morphology, dysfunctions in functional connectivity networks, and alterations in the organization of white and gray matter. Such structural disruptions contribute to the phenotypic complexity of ASD, reflected in the wide variability of social, language, and behavioral deficits observed among affected individuals (Lim et al., 2022).

Emerging evidence indicates that epigenetic dysfunction, involving DNA methylation and histone deacetylation, contributes to the pathogenesis of ASD, particularly when modulated by environmental risk factors during gestation. These epigenetic mechanisms, essential for gene expression regulation, control transcriptional activity: methylation in CpG islands represses transcription under hypermethylation conditions and favors it under hypomethylation conditions. The dysregulation of these processes can result in the inappropriate activation or inhibition of genes crucial for neural development, affecting brain function at both structural and functional levels (Genovese and Butler, 2023).

Histone deacetylation, promoted by histone deacetylases (HDACs), induces chromatin compaction and reduces DNA accessibility, resulting in impaired transcription of genes crucial for the formation and maintenance of excitatory glutamatergic and inhibitory GABAergic synapses. Consequently, alterations in these processes affect brain regions central to the pathophysiology of ASD, such as the cingulate gyrus, amygdala, striatum (caudate-putamen), and cerebellum, compromising neural circuits associated with social cognition, emotional regulation, motor control, and language development (Torres et al., 2023).

Additionally, copy number variations (CNVs) represent an important genetic mechanism associated with ASD pathology. These alterations, which involve deletions or duplications of DNA segments the most frequently observed mutations can be recurrent or non-recurrent. When located in critical regions, such as the 16p11.2 locus, CNVs are often related to ASD, significantly impacting biological functioning (Zhang et al., 2024).

Besides CNV variations, it is estimated that at least 5% of ASD cases are related to single nucleotide polymorphisms (SNPs) in genes such as NLGN3, NLGN4, NRXN1, MECP2, SHANK3, FMR1, TSC1/2, and UBE3A. Approximately 10% of identified CNVs directly affect protein coding through gene duplications or deletions (Jiang et al., 2022).

The complexity of ASD, as well as the mutations associated with it, is not due to the isolated action of specific genetic alterations, but rather to the convergence of these mutations in common cellular and molecular pathways. These pathways are particularly related to fundamental processes such as gene transcription and translation, epigenetic mechanisms, immune and inflammatory responses, as well as synapse formation and functionality (Jiang et al., 2022).

Synapse formation and maturation are essential for the development of neural circuits, with synaptic dysfunction being a potential pathogenic factor involved in neurodevelopmental disorders. Therefore, the role of genes involved in this function has gained significant attention, including mutations in genes such as MECP2, SHANK, FMR1 (Zhang et al., 2024).

The FMR1 gene, whose mutation is causally associated with fragile X syndrome, is also considered the primary known genetic cause of autism. This syndrome is caused by the silencing of the FMR1 gene due to hypermethylation of a trinucleotide CGG repeat sequence located in the gene’s promoter region, preventing its normal expression. The FMRP protein, which is crucial for proper neuronal function, binds to RNA and plays key roles in regulating protein translation in neurons, RNA stability, response to DNA damage, and modulation of ion channels processes essential for homeostasis and neuronal communication (Milla et al., 2023).

The MECP2 gene, on the other hand, encodes a transcriptional regulatory protein essential for neuronal development and function. Mutations in this gene are the leading cause of Rett syndrome, although they have also been associated with autism and neonatal encephalopathy (Gonzales and LaSalle, 2010). Expressed in neural and glial stem cells, MECP2 has higher expression in post-mitotic neurons. Its main function is to modulate gene expression by regulating chromatin structure, acting both as a repressor and an activator, depending on the cellular context. The most critical mutations occur in the MBD and NID domains, impairing interaction with the NCOR1/SMRT complex and compromising the formation of nuclear condensates, key mechanisms for regulating gene activity in the brain (D’Mello, 2021).

Among the SHANK gene family including SHANK1, SHANK2, and SHANK3 the latter is considered one of the promising candidates in the etiology of ASD (Zhang et al., 2024). The SHANK3 gene encodes a scaffolding protein located at the postsynaptic density, crucial for synaptic development, function, and plasticity, coordinating the assembly of macromolecular signaling complexes (Mei et al., 2016). In murine models, the deletion of the SHANK3 gene demonstrated repetitive behaviors, signs of anxiety, and impairments in social interactions features frequently observed in individuals with ASD (Peça et al., 2011).

These genes associated with ASD and specific genetic syndromes directly impact synaptic function. The FMR1 gene regulates the translation of mRNAs related to synaptic components such as glutamate receptors, PSD95, SHANKs, and neurexins. Its dysfunction leads to deregulated translation of targets like NLGN1 and NLGN3, compromising the organization of the postsynaptic membrane and increasing the fluidity of receptors such as mGluR5. MECP2 deletion, in turn, causes delays in neuronal development, reduced synaptic plasticity, and failures in excitatory transmission. However, its overexpression also compromises synaptic integrity, as seen in MECP2 duplication syndrome, which presents autistic phenotypes and alterations in brain structures such as the hippocampus and thalamus. Mutations in SHANK3 are related to Phelan-McDermid syndrome, resulting in reduced postsynaptic proteins (Zhang et al., 2024). Details of genes are summarized in the Table 1.

**Table 1:** Genes and proteins associated with ASD and their roles in brain development.

Gene	Main function	Role in brain development	Alterations observed	References
CALB1	A calcium-binding protein, part of a group of cytosolic proteins that modulate intracellular signals.	Regulates calcium, which is essential for synaptic signaling and maintaining the excitation/inhibition balance.	May be involved in synaptic connectivity and excitation/inhibition mechanisms, often altered in individuals with ASD.	Tavares et al. (2025) and Baimbridge et al. (1992)
MECP2	The MeCP2 (methyl-CpG-binding protein 2) acts as a regulator of neural development.	Contributes to the maturation and maintenance of neurons by influencing dendritic and axonal growth.	Alterations in this gene can lead to abnormalities in the growth of dendrites and axons, impairing proper neuronal development. Mutations in this gene are associated with Rett syndrome.	D’Mello (2021) and Wen et al. (2017)
SHANK3	A synaptic structural protein enriched in the postsynaptic density of excitatory synapses.	Involved in the formation, maturation, and maintenance of synapses.	Mutations in SHANK3 are associated with Phelan-McDermid syndrome (22q13.3 deletion) and autism, due to symptoms such as impaired social interaction and repetitive behaviors observed in animal models.	Uchino and Waga (2013)
FMR1	Encodes the FMRP protein, which is essential for brain development and function.	FMRP regulates the translation of proteins at synapses, influencing synaptic plasticity.	Mutations in FMR1 cause Fragile X syndrome, which is the most common monogenic cause associated with ASD.	Fyke and Velinov (2021)

It is important to consider that there is functional and spatial convergence, revealing that risk genes for ASD are predominantly active during the mid-fetal period, between the 10th and 24th week post-conception. This finding suggests that genetic alterations associated with ASD may impact neuronal circuits during critical stages of brain development, particularly in the prefrontal cortex. These findings reinforce the hypothesis that ASD has an early genetic origin, highlighting the importance of investigating the molecular and cellular processes that occur during this period (Willsey et al., 2013).

This reasoning also supports other discoveries, such as the theory of anatomical anomalies, which suggests that the brain alterations observed in children with ASD are not acquired through environmental factors but occur during the prenatal period, becoming evident in the child’s first year of life (Courchesne et al., 2011).

**Figure 2:**
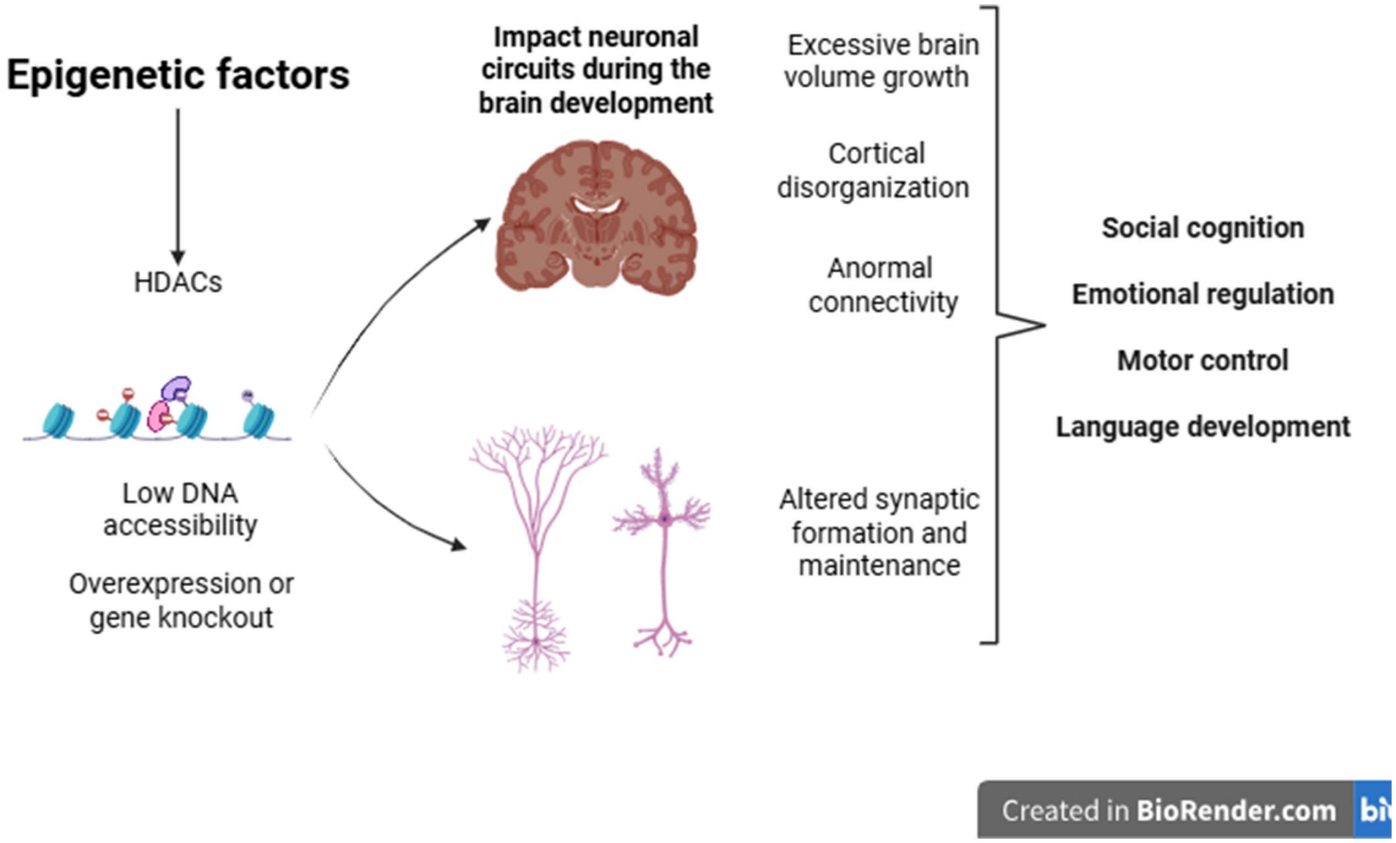
Epigenetic factors during development can cause changes in gene expression in genes related to various cellular processes, such as the formation and maintenance of synapses. Structural brain alterations may also help explain the clinical heterogeneity observed in individuals with ASD.

In addition to the genetic basis, epigenetic factors also contribute to the onset of the condition. These influences result in cellular and morphological changes that affect the brain in a heterogeneous manner. Among these changes, notable ones include abnormalities in brain growth, cortical disorganization, changes in neural connectivity, and synaptic alterations. Such changes provide important evidence for understanding the behavioral and clinical heterogeneity frequently observed in individuals with ASD (Pardo and Eberhart, 2007) (Figure 2).

Thus, considering the genetic, clinical, and behavioral heterogeneity of ASD, there is a challenge in creating universal diagnostic and therapeutic approaches. Advances such as genomic sequencing and the use of functional models have allowed the identification of new risk genes and the elucidation of biological mechanisms related to synaptic function, chromatin remodeling, and neuroinflammation. Despite these discoveries, their clinical application remains limited due to the complexity of gene–environment interactions and the variable expressivity of risk alleles. However, the clinical application of these findings remains restricted, and more longitudinal studies are needed for the development of more precise and individualized strategies (Satterstrom et al., 2020).

## 4 Conclusion

The findings of this review converge on two essential aspects. First, genetic and morphological advances are fundamental for a more comprehensive understanding of the disorder, providing important foundations for understanding its underlying mechanisms. Second, although these results are relevant, they remain incipient and cannot explain the entire heterogeneity involved in ASD, highlighting the need for further investigations.

Despite advances in genetics and epigenetics, challenges persist in clinical application, such as identifying reliable biomarkers and developing individualized interventions. The integration of emerging technologies and interdisciplinary approaches may clarify genetic, epigenetic, and environmental interactions, as well as reveal neurobiological mechanisms, potentially aiding in personalized diagnostics and therapies.

## References

[ref1] American Psychiatric Association (2021). Manual diagnóstico e estatístico e transtornos mentais: DSM-5. Porto Alegre, Brazil: Artmed.

[ref2] Bai D. Yip B. H. K. Windham G. C. Sourander A. Francis R. Yoffe R.. (2019). Association of genetic and environmental factors with Autism in a 5-country cohort. JAMA Psychiatry 76, 1035–1043. doi: 10.1001/jamapsychiatry.2019.1411, PMID: 31314057 PMC6646998

[ref3] Baimbridge K. G. Celio M. R. Rogers J. H. (1992). Calcium-binding proteins in the nervous system. Trends Neurosci. 15, 303–308. doi: 10.1016/0166-2236(92)90081-i, PMID: 1384200

[ref4] Colvert E. Tick B. McEwen F. Stewart C. Curran S. R. Woodhouse E.. (2015). Heritability of autism Spectrum disorder in a UK population-based twin sample. JAMA Psychiatry 72, 415–423. doi: 10.1001/jamapsychiatry.2014.3028, PMID: 25738232 PMC4724890

[ref5] Courchesne E. Campbell K. Solso S. (2011). Brain growth across the life span in autism: age-specific changes in anatomical pathology. Brain Res. 1380, 138–145. doi: 10.1016/j.brainres.2010.09.101, PMID: 20920490 PMC4500507

[ref6] D’Mello S. R. (2021). MECP2 and the biology of MECP2 duplication syndrome. J. Neurochem. 159, 29–60. doi: 10.1111/jnc.15331, PMID: 33638179

[ref7] da Cruz Fontes B. M. de Souza C. B. (2022). Transtorno do espectro autista (TEA): da classificação genética ao diagnóstico molecular. SaBios-Rev. Saúde Biol. 17, 1–9. doi: 10.54372/sb.2022.v17.3405

[ref8] Falcone C. Mevises N.-Y. Hong T. Dufour B. Chen X. Noctor S. C.. (2021). Neuronal and glial cell number is altered in a cortical layer-specific manner in autism. Autism 25, 2238–2253. doi: 10.1177/13623613211014408, PMID: 34107793 PMC9762515

[ref9] Fyke W. Velinov M. (2021). FMR1 and autism, an intriguing connection revisited. Genes (Basel) 12:1218. doi: 10.3390/genes12081218, PMID: 34440392 PMC8394635

[ref10] Genovese A. Butler M. G. (2023). The autism spectrum: behavioral, psychiatric and genetic associations. Genes 14:677. doi: 10.3390/genes14030677, PMID: 36980949 PMC10048473

[ref11] Goldmann J. M. Veltman J. A. Gilissen C. (2019). De novo mutations reflect development and aging of the human germline. Trends Genet. 35, 828–839. doi: 10.1016/j.tig.2019.08.005, PMID: 31610893

[ref12] Gonzales M. L. LaSalle J. M. (2010). The role of MeCP2 in brain development and neurodevelopmental disorders. Curr. Psychiatry Rep. 12, 127–134. doi: 10.1007/s11920-010-0097-7, PMID: 20425298 PMC2847695

[ref13] Hallmayer J. Cleveland S. Torres A. Phillips J. Cohen B. Torigoe T.. (2011). Genetic heritability and shared environmental factors among twin pairs with autism. Arch. Gen. Psychiatry 68, 1095–1102. doi: 10.1001/archgenpsychiatry.2011.76, PMID: 21727249 PMC4440679

[ref14] Hazlett H. C. Poe M. Gerig G. Smith R. G. Provenzale J. Ross A.. (2005). Magnetic resonance imaging and head circumference study of brain size in autism: birth through age 2 years. Arch. Gen. Psychiatry 62, 1366–1376. doi: 10.1001/archpsyc.62.12.1366, PMID: 16330725

[ref15] Hazlett H. C. Poe M. D. Gerig G. Styner M. Chappell C. Smith R. G.. (2011). Early brain overgrowth in autism associated with an increase in cortical surface area before age 2 years. Arch. Gen. Psychiatry 68, 467–476. doi: 10.1001/archgenpsychiatry.2011.39, PMID: 21536976 PMC3315057

[ref16] Hyman S. L. Levy S. E. Myers S. M. Kuo D. Z. Apkon S. Davidson L. F.. (2020). Identification, evaluation, and Management of Children with Autism Spectrum Disorder. Pediatrics 145:e20193447. doi: 10.1542/peds.2019-3447, PMID: 31843864

[ref17] Jiang C.-C. Lin L.-S. Long S. Ke X.-Y. Fukunaga K. Lu Y.-M.. (2022). Signalling pathways in autism spectrum disorder: mechanisms and therapeutic implications. Signal Transduct. Target. Ther. 7:229. doi: 10.1038/s41392-022-01081-0, PMID: 35817793 PMC9273593

[ref18] Lamanna J. Meldolesi J. (2024). Autism Spectrum disorder: brain areas involved, neurobiological mechanisms, diagnoses and therapies. Int. J. Mol. Sci. 25:2423. doi: 10.3390/ijms25042423, PMID: 38397100 PMC10889781

[ref19] Levin A. R. Nelson C. A. (2015). Inhibition-based biomarkers for autism Spectrum disorder. Neurotherapeutics 12, 546–552. doi: 10.1007/s13311-015-0350-1, PMID: 25813603 PMC4489951

[ref20] Lichtenstein P. Carlström E. Råstam M. Gillberg C. Anckarsäter H. (2010). The genetics of autism spectrum disorders and related neuropsychiatric disorders in childhood. Am. J. Psychiatry 167, 1357–1363. doi: 10.1176/appi.ajp.2010.10020223, PMID: 20686188

[ref21] Lim M. Carollo A. Dimitriou D. Esposito G. (2022). Recent developments in autism genetic research: a Scientometric review from 2018 to 2022. Genes 13:1646. doi: 10.3390/genes13091646, PMID: 36140813 PMC9498399

[ref23] Matson J. L. Kozlowski A. M. (2011). The increasing prevalence of autism spectrum disorders. Res. Autism Spectr. Disord. 5, 418–425. doi: 10.1016/j.rasd.2010.06.004

[ref24] Mei T. Forde N. J. Floris D. L. Dell’Acqua F. Stones R. Ilioska I.. (2023). Autism is associated with Interindividual variations of gray and white matter morphology. Biol. Psychiatry Cogn. Neurosci. Neuroimaging 8, 1084–1093. doi: 10.1016/j.bpsc.2022.08.011, PMID: 36075529 PMC7618945

[ref25] Mei Y. Monteiro P. Zhou Y. Kim J.-A. Gao X. Fu Z.. (2016). Adult restoration of Shank3 expression rescues selective autistic-like phenotypes. Nature 530, 481–484. doi: 10.1038/nature16971, PMID: 26886798 PMC4898763

[ref26] Milla L. A. Corral L. Rivera J. Zuñiga N. Pino G. Nunez-Parra A.. (2023). Neurodevelopment and early pharmacological interventions in fragile X syndrome. Front. Neurosci. 17:1213410. doi: 10.3389/fnins.2023.1213410, PMID: 37599992 PMC10433175

[ref27] Nalin L. M. Matos B. A. De Vieira G. G. Orsolin P. C. (2022). Impactos do diagnóstico tardio do transtorno do espectro autista em adultos. Res. Soc. Dev. 11:e382111638175. doi: 10.33448/rsd-v11i16.38175

[ref28] Nóbrega I. D. S. Teles e Silva A. L. Yokota-Moreno B. Y. Sertié A. L. (2024). The importance of large-scale genomic studies to unravel genetic risk factors for autism. Int. J. Mol. Sci. 25:5816. doi: 10.3390/ijms25115816, PMID: 38892002 PMC11172008

[ref29] Pardo C. A. Eberhart C. G. (2007). The neurobiology of autism. Brain Pathol. 17, 434–447. doi: 10.1111/j.1750-3639.2007.00102.x, PMID: 17919129 PMC8095519

[ref30] Parikshak N. N. Luo R. Zhang A. Won H. Lowe J. K. Chandran V.. (2013). Integrative functional genomic analyses implicate specific molecular pathways and circuits in autism. Cell 155, 1008–1021. doi: 10.1016/j.cell.2013.10.031, PMID: 24267887 PMC3934107

[ref31] Peça J. Feliciano C. Ting J. T. Wang W. Wells M. F. Venkatraman T. N.. (2011). Shank3 mutant mice display autistic-like behaviours and striatal dysfunction. Nature 472, 437–442. doi: 10.1038/nature09965, PMID: 21423165 PMC3090611

[ref32] Peterson M. Prigge M. B. D. Bigler E. D. Zielinski B. King J. B. Lange N.. (2021). Evidence for normal extra-axial cerebrospinal fluid volume in autistic males from middle childhood to adulthood. NeuroImage 240:118387. doi: 10.1016/j.neuroimage.2021.118387, PMID: 34260891 PMC8485737

[ref33] Rabelo L. N. Queiroz J. P. G. Castro C. C. M. Silva S. P. Campos L. D. Silva L. C.. (2023). Layer-specific changes in the prefrontal glia/neuron ratio characterizes patches of gene expression disorganization in children with autism. J. Autism Dev. Disord. 53, 3648–3658. doi: 10.1007/s10803-022-05626-8, PMID: 35704132 PMC10084744

[ref34] Roy D. Uddin L. Q. (2021). Atypical core-periphery brain dynamics in autism. Netw. Neurosci. 5, 295–321. doi: 10.1162/netn_a_00181, PMID: 34189366 PMC8233106

[ref35] Satterstrom F. K. Kosmicki J. A. Wang J. Breen M. S. De Rubeis S. An J.-Y.. (2020). Large-scale exome sequencing study implicates both developmental and functional changes in the neurobiology of autism. Cell 180, 568–584.e23. doi: 10.1016/j.cell.2019.12.036, PMID: 31981491 PMC7250485

[ref36] Stoner R. Chow M. L. Boyle M. P. Sunkin S. M. Mouton P. R. Roy S.. (2014). Patches of disorganization in the neocortex of children with autism. N. Engl. J. Med. 370, 1209–1219. doi: 10.1056/NEJMoa1307491, PMID: 24670167 PMC4499461

[ref37] Tavares É. A. F. de Souza D. L. S. da Silva Gomes F. T. Holanda M. V. F. Oliveira R. F. Paiva K. M.. (2025). Calcium-binding proteins in the autistic brain-potential links to symptom development. Int. J. Dev. Neurosci. 85:e10412. doi: 10.1002/jdn.10412, PMID: 39777736

[ref38] Thapar A. Rutter M. (2021). Genetic advances in autism. J. Autism Dev. Disord. 51, 4321–4332. doi: 10.1007/s10803-020-04685-z, PMID: 32940822 PMC8531042

[ref39] Tick B. Bolton P. Happé F. Rutter M. Rijsdijk F. (2016). Heritability of autism spectrum disorders: a meta-analysis of twin studies. J. Child Psychol. Psychiatry 57, 585–595. doi: 10.1111/jcpp.12499, PMID: 26709141 PMC4996332

[ref40] Torres G. Mourad M. Iqbal S. Moses-Fynn E. Pandita A. Siddhartha S. S.. (2023). Conceptualizing epigenetics and the environmental landscape of autism Spectrum disorders. Genes 14:1734. doi: 10.3390/genes14091734, PMID: 37761876 PMC10531442

[ref41] Uchino S. Waga C. (2013). SHANK3 as an autism spectrum disorder-associated gene. Brain Dev. 35, 106–110. doi: 10.1016/j.braindev.2012.05.013, PMID: 22749736

[ref42] Varcin K. J. Nelson C. A. (2016). A developmental neuroscience approach to the search for biomarkers in autism spectrum disorder. Curr. Opin. Neurol. 29, 123–129. doi: 10.1097/WCO.0000000000000298, PMID: 26953849 PMC4850909

[ref43] Wen Z. Cheng T.-L. Li G.-Z. Sun S.-B. Yu S.-Y. Zhang Y.. (2017). Identification of autism-related MECP2 mutations by whole-exome sequencing and functional validation. Mol. Autism. 8:43. doi: 10.1186/s13229-017-0157-5, PMID: 28785396 PMC5543534

[ref44] Willsey A. J. Sanders S. J. Li M. Dong S. Tebbenkamp A. T. Muhle R. A.. (2013). Coexpression networks implicate human midfetal deep cortical projection neurons in the pathogenesis of autism. Cell 155, 997–1007. doi: 10.1016/j.cell.2013.10.020, PMID: 24267886 PMC3995413

[ref45] Xie Y. Sun J. Man W. Zhang Z. Zhang N. (2023). Personalized estimates of brain cortical structural variability in individuals with autism spectrum disorder: the predictor of brain age and neurobiology relevance. Mol. Autism. 14:27. doi: 10.1186/s13229-023-00558-1, PMID: 37507798 PMC10375633

[ref46] Zhang Y. Tang R. Hu Z.-M. Wang X.-H. Gao X. Wang T.. (2024). Key synaptic pathology in autism Spectrum disorder: genetic mechanisms and recent advances. J. Integr. Neurosci. 23:184. doi: 10.31083/j.jin2310184, PMID: 39473158

